# Supplementary material for: A UMLS-based spell checker for natural language processing in vaccine safety
Source: BMC Med Inform Decis Mak. 2007 Feb 12;7:3. doi: 10.1186/1472-6947-7-3 (PMC1805499; doi:10.1186/1472-6947-7-3)
Supplement: Additional file 13 — RAP application directory. Sets up directory for RAP (RDF application for PHP) source code [file 1472-6947-7-3-S13.gz › rap/doc/phpdoc/classtrees_utility.html]

xml version="1.0" encoding="iso-8859-1"?


## Root class IterFind

- IterFind

## Root class Object

- Object
  - DbStore
  - FindIterator
  - GRDDLParser
  - Model
    - DbModel
      - NamedGraphDb
    - MemModel
      - InfModel
        - InfModelB
        - InfModelF
      - NamedGraphMem
  - N3Parser
  - N3Serializer
  - Node
    - Literal
      - ResLiteral
    - Resource
      - BlankNode
      - ResResource
        - OntResource
          - Individual
          - OntClass
          - OntProperty
        - ResContainer
          - ResAlt
          - ResBag
          - ResSeq
        - ResList
        - ResProperty
  - NTripleSerializer
  - RdfParser
  - RdfSerializer
  - RDFUtil
  - RdqlEngine
    - RdqlDbEngine
    - RdqlMemEngine
  - RdqlParser
  - RdqlResultIterator
  - Statement
    - InfStatement
  - StatementIterator

Documentation generated on Thu, 7 Jul 2005 13:41:08 +0200 by phpDocumentor 1.3.0RC3
